# Plant-derived compounds stimulate the decomposition of organic matter in arctic permafrost soils

**DOI:** 10.1038/srep25607

**Published:** 2016-05-09

**Authors:** Birgit Wild, Norman Gentsch, Petr Čapek, Kateřina Diáková, Ricardo J. Eloy Alves, Jiři Bárta, Antje Gittel, Gustaf Hugelius, Anna Knoltsch, Peter Kuhry, Nikolay Lashchinskiy, Robert Mikutta, Juri Palmtag, Christa Schleper, Jörg Schnecker, Olga Shibistova, Mounir Takriti, Vigdis L. Torsvik, Tim Urich, Margarete Watzka, Hana Šantrůčková, Georg Guggenberger, Andreas Richter

**Affiliations:** 1 Department of Microbiology and Ecosystem Science, University of Vienna, Vienna, Austria; 2 Austrian Polar Research Institute, Vienna, Austria; 3 Department of Earth Sciences, University of Gothenburg, Gothenburg, Sweden; 4 Institute of Soil Science, Leibniz Universität Hannover, Hannover, Germany; 5 Department of Ecosystem Biology, University of South Bohemia, České Budějovice, Czech Republic; 6 Department of Ecogenomics and Systems Biology, University of Vienna, Vienna, Austria; 7 Department of Biology, Centre for Geobiology, University of Bergen, Bergen, Norway; 8 Department of Bioscience, Center for Geomicrobiology, Aarhus, Denmark; 9 Department of Physical Geography, Stockholm University, Stockholm, Sweden; 10 Central Siberian Botanical Garden, Siberian Branch of Russian Academy of Sciences, Novosibirsk, Russia; 11 Soil Science and Soil Protection, Martin-Luther-University Halle-Wittenberg, Halle (Saale), Germany; 12 Department of Natural Resources and the Environment, University of New Hampshire, Durham, NH, USA; 13 VN Sukachev Institute of Forest, Siberian Branch of Russian Academy of Sciences, Krasnoyarsk, Russia; 14 Lancaster Environment Centre, Lancaster University, Lancaster, UK; 15 Institute of Microbiology, Ernst-Moritz-Arndt University, Greifswald, Germany

## Abstract

Arctic ecosystems are warming rapidly, which is expected to promote soil organic matter (SOM) decomposition. In addition to the direct warming effect, decomposition can also be indirectly stimulated via increased plant productivity and plant-soil C allocation, and this so called “priming effect” might significantly alter the ecosystem C balance. In this study, we provide first mechanistic insights into the susceptibility of SOM decomposition in arctic permafrost soils to priming. By comparing 119 soils from four locations across the Siberian Arctic that cover all horizons of active layer and upper permafrost, we found that an increased availability of plant-derived organic C particularly stimulated decomposition in subsoil horizons where most of the arctic soil carbon is located. Considering the 1,035 Pg of arctic soil carbon, such an additional stimulation of decomposition beyond the direct temperature effect can accelerate net ecosystem C losses, and amplify the positive feedback to global warming.

Plant productivity in the Arctic is stimulated by rising temperatures^1^,^2^, which implies not only an increased uptake of CO_2_ from the atmosphere by plants, but also an increased transfer of organic compounds from plants to the soil, e.g., as root exudates and root litter^3^. Such an increased input of plant-derived compounds can reduce the microbial decomposition of native SOM by providing soil microorganisms with additional, easily degradable C and N sources that thus decrease the microbial dependence on the more complex substrates of native SOM (“negative priming”)^4^. On the other hand, increased C and N availability can also stimulate SOM decomposition (“positive priming”), since additional N may promote the synthesis of extracellular enzymes that break down polymeric compounds of SOM^5^, whereas additional C may provide microorganisms with energy that facilitates the decomposition of energy-poor SOM compounds^6^,^7^. Additional C can also stimulate microbial growth in general, and thus lead to higher microbial N demand and higher microbial N mining, i.e., to a higher microbial decomposition of SOM to get access to N (refs. 8,9).

Studies on an ecosystem level suggest that an increased allocation of plant-derived organic compounds into the soil with warming can indeed stimulate the decomposition of native SOM. For instance in the European sub-Arctic, significantly smaller soil organic C (SOC) stocks have been observed in a forest than in an adjacent tundra, indicating that the transition from tundra to forest with warming can lead to a net loss of C from the soil, which in this case even exceeded the higher plant C stocks in the forest^10^. In contrast, in an Alaskan tussock tundra, ten years of warming stimulated plant primary production, but did not lead to a net change in SOC stocks^11^. This variability in the effect of plant-derived compounds on SOM decomposition might be related to differences in the distribution of SOM through the soil profile, and in the susceptibility of its decomposition to changes in organic C and N availability.

For instance, SOM in the top soil layer (further termed “organic topsoil”) mainly consists of poorly decomposed plant material with a high content of easily degradable C sources such as cellulose, but a comparatively low content of N. The decomposition of organic topsoil material is therefore expected to be rather insensitive to an increased input of organic C, but might be strongly affected by changes in the availability of organic or inorganic N. In line with a predominant N control on SOM decomposition in the organic topsoil, inorganic N addition has been found to stimulate the decomposition of organic topsoil material^12^,^13^, whereas organic C addition had hardly any effect^14^,^15^. In contrast to the organic topsoil, SOM in the subsoil is partly bound to soil minerals, has been repeatedly processed by soil microorganisms, and is characterized by low C/N ratios. The decomposition of mineral subsoil material has been found to strongly respond to the addition of organic C, with more than a doubling of SOC mineralization rates^15^. Consequently, particularly pronounced effects of increased plant C input on the decomposition of SOM in mineral subsoil horizons of arctic permafrost soils have been suggested^15^.

Furthermore, arctic permafrost soils are often characterized by a mixing of soil horizons due to freeze-thaw processes that lead to the burial of poorly decomposed organic material from the topsoil into the mineral subsoil (“cryoturbation”; for a recent review see ref. 16). Cryoturbated material shows particularly low decomposition rates^15^,^17^, and although it is located in the subsoil, decomposition rates might depend not on C, but rather on N availability, as indicated by a stimulation of decomposition after addition of organic N, but not of C alone^15^. Since arctic soils store about 1,035 Pg of organic C, with more than 80% of that in horizons deeper than 30 cm (ref. 18), understanding the controls over SOM decomposition and its response to changes in C and N availability across soil horizons is crucial for predicting C losses from arctic ecosystems in a future climate.

In this study, we provide first mechanistic insight into the susceptibility of SOM decomposition in arctic permafrost soils to an increased input of plant-derived compounds, such as by enhanced root litter production in a warmer climate. For 119 individual soil samples derived from four locations across the Siberian Arctic (Fig. 1, Table 1) and from five soil horizon categories (organic topsoil, mineral topsoil, mineral subsoil and cryoturbated material from the active layer, and mineral subsoil material from the upper permafrost), we simulated such an increased input of plant-derived compounds in a laboratory experiment, by amending soil samples with ^13^C-labelled plant polymers, either cellulose or protein. Expecting a transition from N to C limitation of the microbial community with progressing SOM decomposition, we hypothesized that (1) SOM mineralization in organic topsoils and cryoturbated material would be affected by organic N, but not organic C, and would hence be stimulated by protein, but not by cellulose, and that (2) SOM mineralization in mineral soil horizons would be affected by organic C irrespective of the N content of the substrate, and would hence be stimulated by both cellulose and protein. After the addition of the respective substrate, we incubated the soil samples for 25 weeks and monitored soil respiration, distinguishing between substrate-derived (i.e., ^13^C-enriched) and SOC-derived (i.e., non-enriched) CO_2_. At the end of the incubation, we finally determined microbial biomass and microbial substrate use efficiency.

## Results

### Characterization of Soil Organic Matter

From organic to mineral topsoils and further to mineral subsoils, organic C content, total N content, as well as C/N ratios decreased, and δ^13^C values increased, reflecting the proceeding decomposition state of SOM with depth (Table 2). Cryoturbated material was sampled at a similar depth as the mineral subsoil, but was characterized by more abundant and less decomposed SOM, with organic C and total N contents, C/N ratios and δ^13^C values in the range of the mineral topsoil. Mineral subsoils from the permafrost were similar to mineral subsoils from the active layer in terms of organic C, total N and C/N ratios, but had significantly higher δ^13^C values.

### Soil Organic Carbon Mineralization

Based on cumulative SOC-derived respiration (Supplementary Fig. S1), we calculated the amount of SOC lost by mineralization during the 25 weeks of incubation. In the control samples, on average 6.1 ± 0.6% (mean ± standard error) of SOC were lost from organic topsoils, 4.3 ± 0.5% from mineral topsoils, and 2.8 ± 0.5% from mineral subsoils (Fig. 2). Hence, SOC loss decreased with increasing decomposition state of SOM. Although cryoturbated material was similar to mineral topsoil material in terms of organic C and total N content, C/N ratio and δ^13^C, it showed particularly low C mineralization rates, with only 1.6 ± 0.1% SOC lost during the incubation. Permafrost samples, in contrast, were characterized by SOC losses in the range of the organic topsoil (5.8 ± 1.2%).

Addition of cellulose or protein generally stimulated SOM decomposition, as indicated by an increase in the mineralization of native SOC (Fig. 3). Addition of cellulose had no significant effect in organic topsoils, whereas addition of protein led to a significant increase in SOC mineralization (response ratio (RR) = 1.51, corresponding to an average increase by 51%). A decrease in SOC mineralization was only observed in few of the organic topsoil samples, with response ratios of less than 0.80 in 18% (cellulose) and 11% (protein) of the incubated samples. This effect was likely due to a switch of microorganisms from SOM to cellulose or protein as substrate. Substrate replacement was negligible in the other horizons (Supplementary Table S2).

In mineral topsoils, mineral subsoils, and cryoturbated material, cellulose addition significantly increased SOC mineralization (RR = 1.22, 1.31, and 1.22, respectively), whereas the stimulation in permafrost material was not significant (RR = 1.23; Fig. 3). Effects of protein were stronger than of cellulose and significant in all horizons, with response ratios of 1.41 and 2.20 in mineral topsoils and mineral subsoils, 2.09 in cryoturbated material and 1.63 in permafrost material. The amount of SOC additionally mineralized after addition of cellulose or protein exceeded the respective biomass C pools by average factors of 2.0 (cellulose; organic topsoil not included) and 8.3 (protein), indicating that the additional SOC mineralization was not caused by an accelerated turnover of the microbial biomass (“apparent priming”), but by an enhanced decomposition of SOM (“real priming”)^7^.

In mineral subsoils from the active layer and the permafrost, we further found significant correlations between the responses of SOC mineralization to cellulose and protein addition (Table 3), i.e., samples that responded to cellulose also responded to protein. Similar correlations were not observed in the other horizons.

Over the 25 weeks of incubation at 15 °C, the stimulation of SOM decomposition by cellulose and protein resulted in additional losses of native SOC of up to 1.0% SOC (cellulose) and 1.3–2.8% SOC (protein), depending on soil horizon (Fig. 2). Based on this incubation, we estimated SOC mineralization across a whole growing-season. Assuming a four-month season where soils are thawed^19^ and plants are productive^20^, as well as soil temperatures as measured in the field during sampling, the results of our laboratory experiment correspond to SOC losses of 0.7–2.7% without additional input of substrates. An input of cellulose or protein as simulated in our experiment would thus induce additional losses of up to 0.6% (cellulose) and 0.5–1.1% (protein) of native SOC (Table 4).

### Microbial Growth and Substrate Utilization

In the control samples, microbial biomass decreased with soil depth following the decrease in SOM content. Calculated per unit SOC, microbial biomass was significantly lower in cryoturbated material than in the other soil horizons (Supplementary Table S3). The addition of cellulose led to a significant increase in microbial biomass in mineral topsoils (RR = 1.64), and the addition of protein had a similar effect in mineral topsoils (RR = 1.57), as well as in cryoturbated material (RR = 1.48; Fig. 4). In the other cases, substrate additions did not induce significant changes in microbial biomass.

Similar to the responses in SOC mineralization, we found significant correlations between the effects of cellulose addition and protein addition on microbial biomass in mineral subsoils of the active layer and the permafrost (Table 3). We further tested if responses of microbial biomass were connected to responses of SOC mineralization, but did not find any significant correlation, neither for cellulose nor for protein, and neither for individual horizons nor across all samples.

Since microbial biomass was only measured at the end of the incubation, our data do not consider potential transient peaks in microbial biomass, e.g., shortly after substrate addition. However, even at the end of the incubation after 25 weeks, we found ongoing mineralization of substrate-derived C (Supplementary Fig. S1) that could have stimulated microbial growth. Considering the lack of significant substrate effects on microbial biomass in mineral subsoils of active layer and permafrost, and the lack of correlation between responses of biomass and decomposition, we argue that the observed increase in SOM decomposition was likely not linked to an increase in microbial biomass.

Based on cellulose- or protein-derived C in cumulative respiration and microbial biomass, we calculated microbial substrate use efficiencies for cellulose and protein over the incubation time. Substrate use efficiencies were generally low, likely due to repeated turnover of the microbial biomass over the long incubation time, but still showed significant differences between substrates and horizons (Fig. 5). For all horizons except the organic topsoils, substrate use efficiencies were significantly higher for cellulose than for protein, and in both treatments, substrate use efficiencies were highest in mineral subsoils from the active layer and the permafrost, i.e., microorganisms allocated more substrate-derived C to growth and less to respiration than in the other horizons. Substrate use efficiencies for cellulose and protein were significantly correlated with the C/N ratios of SOM when calculated across all horizons, with high substrate use efficiency at low C/N (cellulose: p < 0.001, rho = −0.481; protein: p < 0.001, rho = −0.343; Supplementary Table S4). In contrast, we found no significant correlation between SOM stoichiometry or microbial substrate use efficiency and the response of SOC mineralization to substrate addition (Supplementary Table S4).

## Discussion

Arctic soils contain about 1,035 Pg of organic C (ref. 18), more C than in today’s atmosphere. According to recent model estimates, about 15% of this SOC will be lost as CO_2_ or CH_4_ until 2100 as a consequence of rising temperatures and permafrost thaw^21^. Our findings suggest that in addition to these direct temperature effects, SOC losses from arctic soils can be further promoted by changes in the availability of organic C or N, for example due to enhanced root litter production. Our findings further point to mechanistic differences between soil horizons: We found a high susceptibility (1) of organic topsoils to changes in organic N availability, (2) of mineral subsoils in active layer and permafrost to changes in organic C availability, and (3) of mineral topsoils and cryoturbated material to both.

In organic topsoils, the decomposition of native SOM was significantly altered by protein, but not by cellulose (Fig. 3), and we suggest that this effect was linked to protein-derived N. Previous studies on organic topsoil horizons of arctic soils have shown high microbial N use efficiency^22^,^23^ and strong effects of inorganic N additions, including a stimulation of microbial growth^13^ and SOM decomposition^12^,^13^, as well as changes in microbial enzyme production^13^,^24^. Taken together, these studies indicate predominant N limitation of the microbial decomposer community in organic topsoil horizons of arctic permafrost soils and suggest a high susceptibility to changes in the availability of both inorganic and organic N. Increased organic N availability in organic topsoils can result from an increased input of root and leaf litter-derived proteins with rising temperatures^25^, as simulated in our study. Warming additionally promotes a more active decomposer community^26^ and increases the efficiency of extracellular enzymes^27^, and might thus facilitate the depolymerization of proteins and other N-containing polymers into smaller units that can then be taken up by microorganisms and plants. Although plant N demand is also expected to increase with warming, several field studies show higher net N mineralization at higher temperatures and thus point to an overall increase in topsoil N availability^28^,^29^. In our study, an increase in protein availability overall stimulated SOM decomposition in the organic topsoil (Fig. 3), but we also observed a significant reduction at one of the four sites, suggesting that the direction of the response can differ between individual sites (Supplementary Table S5). A similar site-dependency in the response of SOM decomposition has also been observed for inorganic N additions^12^.

We hypothesized that in deeper, mineral soil horizons, the microbial decomposer community would be increasingly limited by low C availability, and our findings support this hypothesis. Below a threshold of about 10% SOC and a C/N ratio of 20, not only protein, but also cellulose addition led to an overall stimulation of SOM decomposition (Supplementary Fig. S2). For mineral subsoils from active layer and permafrost, we further found significant correlations between priming effects by cellulose and protein (Table 3), as well as an efficient incorporation of cellulose- and protein-derived C into the microbial biomass (Fig. 5) that points at high microbial C use efficiency. These findings suggest that not only the effect of cellulose, but also of protein on SOM decomposition was at least partly induced by increased C availability. However, the effects of protein on SOM decomposition in general exceeded those of cellulose. This might have been due to a higher bio-availability of protein- than of cellulose-derived C, or due to the additional N contained in proteins that might have facilitated the synthesis of extracellular enzymes that break down SOM. Considering the low C/N ratio of proteins, such an additional N fertilization effect seems likely also in mineral subsoil horizons of active layer and permafrost.

For mineral topsoils and cryoturbated material, we did not find patterns that would suggest a distinct susceptibility of SOM decomposition to changes in either organic C or N availability, given the independent stimulating effects of both cellulose and protein (Fig. 3, Table 3). Mineral topsoil and cryoturbated material are at an intermediate state of decomposition compared to organic topsoil and mineral subsoil material (Table 2; see also ref. 30), and microbial C versus N limitation might differ strongly between individual soil samples. This variability was not connected to differences in bulk SOM stoichiometry (Supplementary Table S4). For cryoturbated material in particular, previous studies have suggested low bio-availability of the N present^22^, and have shown a strong stimulation of SOM decomposition after addition of organic N, but not organic C alone^15^. Also in this study, effects of protein were by far stronger than those of cellulose. However, our findings suggest that not only low N availability, but also low C availability can constrain the decomposition of cryoturbated material.

With rising temperatures in the Arctic, increases in belowground plant biomass^11^, in root production^31^, and in plant belowground C transfer^32^ have been observed. Our findings indicate that an increased transfer of organic compounds from plants to the soil (e.g., via root litter) can stimulate the decomposition of native SOM especially in deeper soil horizons, i.e., below the organic topsoil. Although the decomposition of SOM in these horizons is constrained by its low quality and by low temperatures, deeper soil horizons showed not only the highest relative increases in SOC mineralization after addition of cellulose or protein (Fig. 3), but also absolute increases in SOC mineralization within the range observed for organic topsoils (Fig. 2). This pattern prevailed even when we considered the decrease in soil temperature with depth under field conditions (Table 4). With the majority of arctic SOC in horizons below 30 cm (ref. 18), an enhanced mineralization of this SOC might strongly affect the C balance of arctic ecosystems, and amplify the positive feedback between permafrost CO_2_ emissions and global warming (e.g., ref. 33). Mineral and cryoturbated horizons of the active layer are estimated to store 360 Pg of organic C (ref. 34), and the projected thawing of the upper permafrost table (active layer deepening) is expected to increase the stocks available for SOM decomposition by another 109 Pg until 2100 (mineral permafrost soil expected to thaw under moderate radiative forcing^34^). Although results from laboratory incubation experiments cannot be directly translated to the field, it is still interesting to note that additional losses of native SOC induced by cellulose or protein input in our experiment would correspond to additional SOC losses in the order of 1.2 Pg (cellulose) or 3.8 Pg (protein) across a four-month growing-season, demonstrating the potential for changes in C cycling by priming in permafrost soils. By comparison, CO_2_ production from fossil fuels accounts for approximately 7.8 Pg C per year^35^.

We emphasize that our extrapolated values likely represent the upper limit for potential additional SOC losses, given the favourable conditions for decomposition and the ample supply of plant-derived compounds in our experiment. The extent to which these potential losses will be realized will depend on abiotic constraints on decomposition (e.g., anoxic conditions, protection by soil aggregates) as well as on quantity and quality of additional plant-derived organic compounds. For instance, deep active layer and current permafrost were most susceptible to an increased availability of plant-derived compounds in our experiment, but are hardly affected by plant roots under field conditions, given that more than 90% of plant roots are currently located in the top 30 cm of the soil^36^. However, as permafrost soils get warmer and the active layer deepens, plants might increasingly access deeper soil horizons to take up nutrients. Our findings suggest that the extent of such changes in plant rooting depth will strongly determine the magnitude of additional SOC losses induced by priming.

While our findings show that a higher availability of plant-derived organic compounds can considerably stimulate SOC mineralization in deeper soil horizons, the consequences for the ecosystem C budget will depend on the balance between additional plant primary production and additional C mineralization, including the direct stimulation of SOM decomposition by soil warming, the stimulation induced by increased plant-soil C allocation, and the decomposition of the additionally produced plant litter itself. Recent studies on an ecosystem scale suggest that both net C sequestration and net C losses might be observed: (1) Net ecosystem C sequestration will then occur where the enhanced CO_2_ fixation by plants exceeds the additional losses by SOC mineralization. Such a case has been observed in a tussock tundra, where ten years of warming promoted belowground plant biomass and microbial activity in the mineral soil horizon, and overall increased organic C storage in this horizon, and in the entire ecosystem^11^. (2) Net C losses from the ecosystem will occur where the additional C mineralization exceeds the additional CO_2_ fixation. Such a case has been suggested for the tree line in the European sub-Arctic, where ecosystem C stocks were lower in a forest than in an adjacent tundra, and where this difference has been specifically attributed to a stimulation of SOC mineralization in the forest due to higher plant-soil C allocation^10^. Also a range of other studies have observed a decrease in arctic ecosystem C storage due to warming^37^,^38^, but contributions of direct stimulating effects on SOM decomposition, effects mediated by increased N availability and effects mediated by increased plant-soil C allocation have not been distinguished. Whether the stimulation of SOM decomposition will overall only reduce the ecosystem C sink strength, or even induce net ecosystem C losses, might thus depend on functional properties of the initially dominant plants and on changes in plant species composition with warming, for instance related to quantity and quality of root litter and root exudates, to rooting depths, or to mycorrhizal associations^10^,^39^.

## Material and Methods

### Soil Sampling

**Figure 1:**
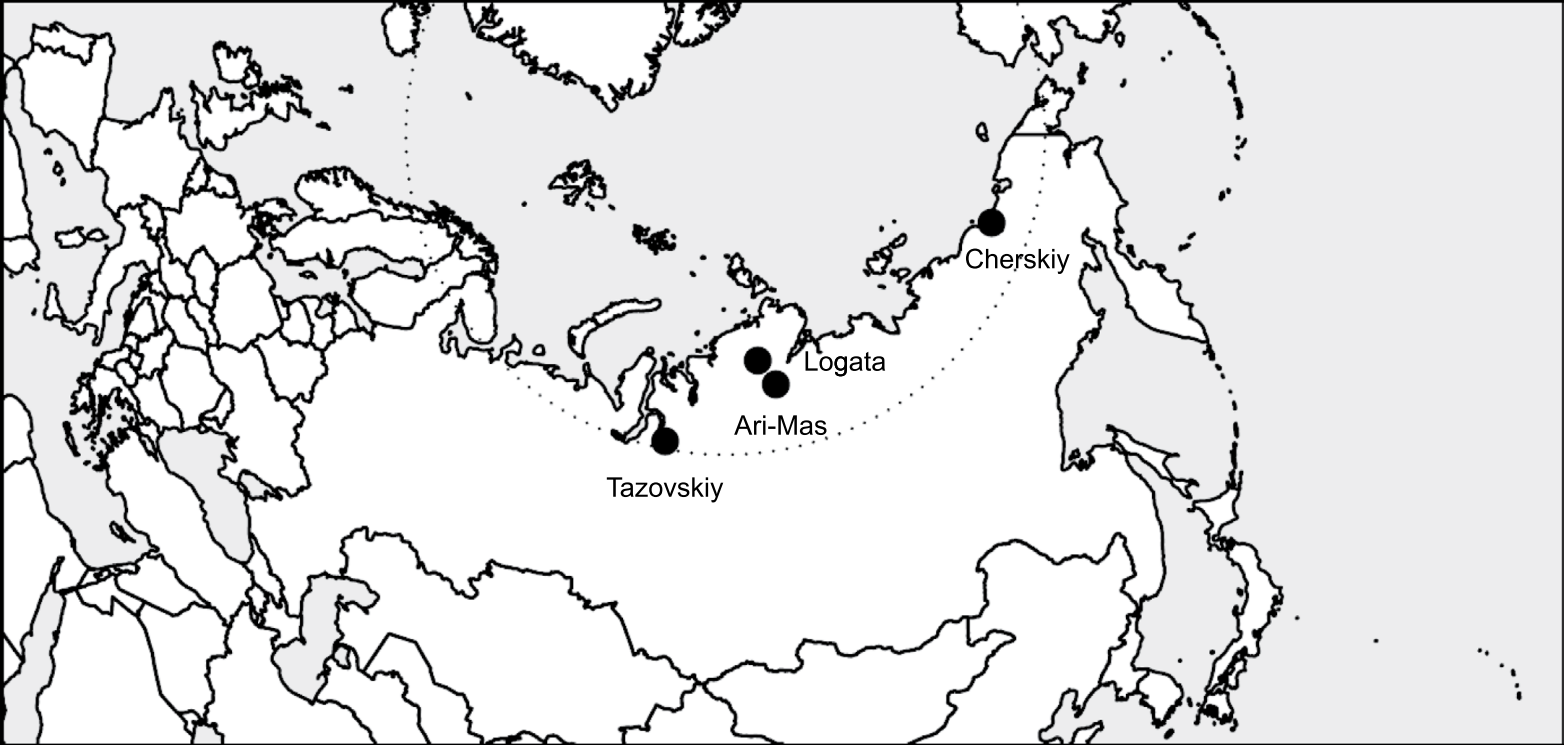
Map of sampling sites across the Siberian Arctic. The dotted line indicates the polar circle. The map was created in R using the packages sp and rworldmap^47^,^49^,^50^.

**Figure 2:**
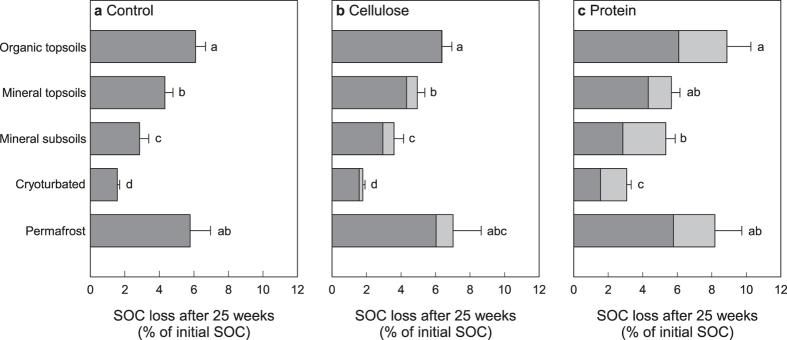
Losses of native SOC from different horizons of arctic permafrost soils after 25 weeks of incubation (dark grey bars). Losses induced by the addition of cellulose or protein in comparison to control samples are indicated in light grey. Bars represent means with standard errors, different letters indicate significant differences between horizons at p < 0.05. See Supplementary Fig. S1 for the development of SOC- and substrate-derived respiration over time.

**Figure 3:**
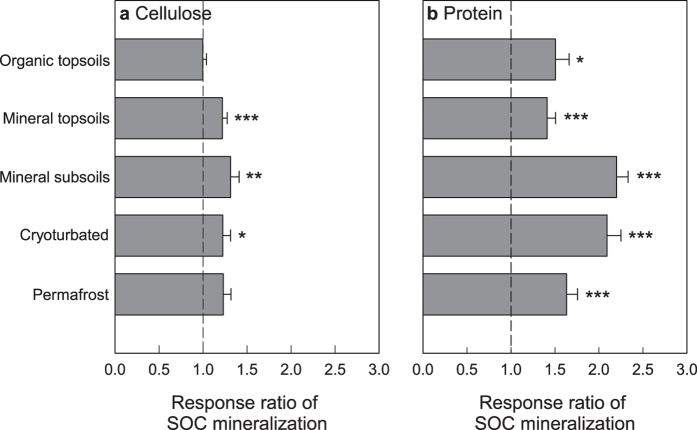
Response of cumulative SOC mineralization in different horizons of arctic permafrost soils to addition of cellulose or protein. Response ratios were calculated as ratios of samples amended with cellulose or protein over control samples. Bars represent means with standard errors, significant differences in SOC mineralization between amended and control samples are indicated (Welch’s paired t-tests; ***p < 0.001; **p < 0.01; *p < 0.05). For response ratios at individual sampling sites see Supplementary Table S5.

**Figure 4:**
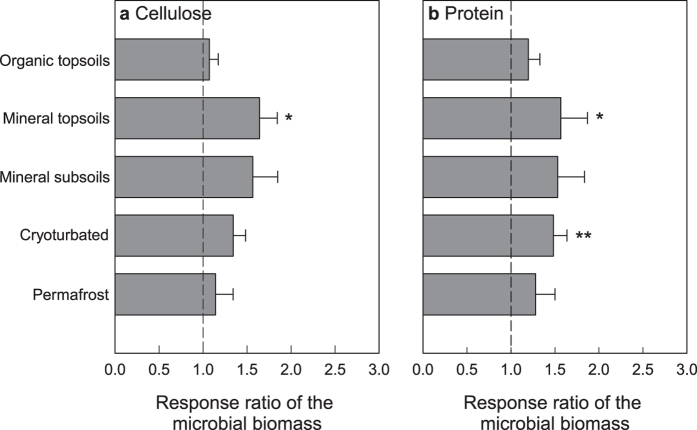
Response of the microbial biomass in different horizons of arctic permafrost soils to addition of cellulose or protein. Response ratios were calculated as ratios of samples amended with cellulose or protein over control samples. Bars represent means with standard errors, significant differences in microbial biomass between amended and control samples are indicated (Welch’s paired t-tests; **p < 0.01; *p < 0.05). For microbial biomass in control samples see Supplementary Table S3, and for response ratios at individual sampling sites see Supplementary Table S5.

**Figure 5:**
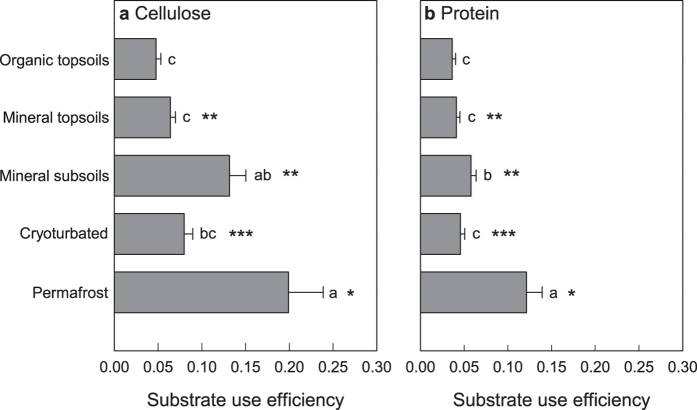
Microbial substrate use efficiency of cellulose- or protein-derived C in different horizons of arctic permafrost soils. Substrate use efficiency was calculated as the ratio of substrate-derived C in microbial biomass over substrate-derived C in biomass and cumulative respiration after 25 weeks of incubation. Bars represent means with standard errors. Significant differences between cellulose and protein treatments are asterisked (***p < 0.001; **p < 0.01; *p < 0.05), significant differences between horizons for cellulose or protein are indicated by different letters (p < 0.05). For data on individual sampling sites see Supplementary Table S6.

Soils for the incubation experiment were sampled at four sites across the Siberian Arctic, in the areas of Cherskiy (Eastern Siberian Arctic), Ari-Mas, Logata (both Central Siberian Arctic), and Tazovskiy (Western Siberian Arctic; Fig. 1). All sampling sites were underlain by continuous permafrost, and samples were taken in the late growing-season at the maximum thaw depth. Sites are described briefly in Table 1, and in detail in ref. 30.

At each study site, we identified two zonal upland tundra vegetation types that were representative for the respective landscape. For each vegetation type, we excavated three soil profiles of 5 m length down to the permafrost table, and sampled soil horizons from these profiles. We additionally sampled the upper part of the permafrost using a steel corer, to a maximum depth of 30 cm from the permafrost table. Soil samples were grouped into five categories: Organic topsoils were O horizons (1), mineral topsoils comprised OA, A and AB horizons (2), and mineral subsoils BC and C horizons from the active layer (3). We further identified pockets of cryoturbated material (Ojj and Ajj) in the active layer that were characterized by a higher SOM content than the surrounding mineral soil; these samples formed a separate category (4). Finally, samples of mineral subsoil from the upper permafrost were classified separately (5). Unless specified otherwise, we refer to mineral subsoils from active layer as “mineral subsoils” and to mineral subsoils from the permafrost as “permafrost”. Directly after sampling, living roots were removed, samples were air-dried, and stored in a dark, cool, and dry place. Air-drying of the soil samples was a prerequisite for sample transport from the remote field sites to the lab, but might have introduced a certain bias by reducing the active microbial community. In order to re-activate the microbial community, we therefore allowed for a pre-incubation period. Two weeks before the start of the experiment, we weighed triplicates of 2.5 g (O and OA horizons), 5 g (Ojj and Ajj horizons) or 10 g soil (A, AB, BC, and C horizons of the active layer, as well as permafrost samples) into glass bottles (headspace 100–130 ml), adjusted water contents to 60% water holding capacity, and loosely plugged the bottles with polyethylene wool. Samples were pre-incubated in the dark at 15 °C. Respiration rates measured after two weeks were similar to rates in fresh samples of organic topsoil, mineral subsoil and cryoturbated material from the Siberian Arctic measured in a previous experiment^15^, and we therefore consider the two week pre-incubation sufficient for the re-establishment of an active native microbial community. This is supported by previous studies showing minor long-term effects of a single drying-rewetting event on microbial community composition and decomposition processes^40^,^41^,^42^,^43^.

Carbon and N content were determined in dried and ground samples with EA-IRMS (Elementar vario MICRO cube EA, Elementar Analysensysteme GmbH, Germany, and Elementar IsoPrime 100 IRMS, IsoPrime Ltd., UK). Sample numbers, sampling depths, average contents of organic C and total N, C/N ratios, and C isotope values are given in Table 2 and Supplementary Table S1.

### Setup of the Incubation Experiment

^13^C-labelled cellulose (Isolife *Cichorium intybus* cellulose, U^13^C, >97 at%) and ^13^C-labelled protein (Sigma-Aldrich algal crude protein extract, U^13^C, 98 at%) were diluted with unlabelled cellulose or protein, respectively, to reach 5 at% ^13^C. Triplicates of the pre-incubated samples were assigned to the three treatments (control, cellulose, protein), and samples assigned for cellulose or protein treatments were amended with 5 at% ^13^C cellulose or ^13^C protein in amounts equivalent to 4% SOC by mixing the dry powder into the soil. Neither cellulose nor protein significantly affected the pH of the soil.

In contrast to monomeric compounds such as glucose and amino acids that can be immediately taken up by soil microorganisms, cellulose and protein have to be broken down by extracellular enzymes before uptake. The addition of cellulose or protein, even of rather high amounts as in this study, therefore leads to only a slight, but persistent increase in organic C and N availability for soil microorganisms, as indicated by the ongoing respiration of cellulose- and protein-derived C even after 25 weeks of incubation (Supplementary Fig. S1), and thus more closely mimics the long-term mobilization of organic C and N during root litter decomposition.

After substrate addition, samples were again plugged with polyethylene wool and incubated at 15 °C for 25 weeks. During the course of the incubation, samples were weighed weekly and water lost by evaporation was replaced with ultrapure water.

### Respiration Measurements

Respiration rates were measured immediately after substrate addition, and after 7, 14, 21, 28, 42, 56, 84, 112 and 175 days. For gas sampling, bottles were closed with rubber plugs and flushed with CO_2_-free air. Gas samples were taken after 24–48 h incubation at 15 °C with gas tight syringes and analysed with gas chromatography (Cherskiy and Tazovsky: Agilent 7820A GC, Agilent Technologies; Ari-Mas and Logata: Shimadzu GC 2014, Shimadzu). CO_2_ concentrations were corrected for the amount of CO_2_ dissociated in the soil solution^44^. To determine the C isotope composition of respired CO_2_, additional gas samples were taken on days 9, 44, 86, and 177 as described above and analysed with a GasBench II system coupled to a Delta V Advantage IRMS (Thermo Scientific). Based on the C isotope composition of respired CO_2_, we distinguished between CO_2_ derived from the added substrate and from SOC using the equations


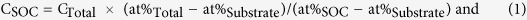



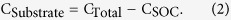


C_Total_, C_SOC_ and C_Substrate_ are total, SOC-derived and substrate-derived CO_2_, and at%_Total_, at%_SOC_ and at%_Substrate_ are isotopic compositions (in at% ^13^C) of total, SOC-derived and substrate-derived CO_2_, respectively. The isotopic composition of SOC-derived CO_2_ was set to the respective value for unamended control samples. Based on the contributions of substrate-derived and SOC-derived CO_2_ to total respiration at these timepoints, we interpolated these values for the other timepoints where ^13^CO_2_ data were not available, and calculated the cumulative amounts of CO_2_ released from the added substrate and from SOC over the course of the incubation. Throughout the text, we use the term “SOC mineralization” exclusively for CO_2_ production from native, unlabelled SOC, excluding C mineralized from added, ^13^C-labelled cellulose or protein.

Since mineral, cryoturbated and permafrost horizons are not likely to experience temperatures of 15 °C under field conditions, we additionally estimated SOC mineralization for soil temperatures typical for the growing-season. Based on field measurements during sampling, we estimated growing-season soil temperatures as 8 °C (organic topsoil), 7.5 °C (mineral topsoil), 4.5 °C (mineral subsoil), and 4.1 °C (cryoturbated material). Permafrost material was frozen at the time of sampling, but since our experiment simulated the exposure of recently thawed permafrost to increased cellulose or protein input, we assumed a temperature of 1.0 °C. We finally estimated SOC mineralization rates and additional SOC losses induced by cellulose and protein for typical growing-season temperatures using Q_10_ values determined for the same set of soil samples in a second incubation experiment (Gentsch *et al.*, unpublished).

### Microbial Biomass and Substrate Use Efficiency

Content and isotopic composition of microbial C were estimated with chloroform-fumigation-extraction^15^,^45^. Soil samples fumigated with chloroform, as well as non-fumigated samples, were extracted with 0.5 M K_2_SO_4_ and dissolved organic C was measured using an HPLC-IRMS system in direct injection mode against sucrose standards^46^. Microbial C content was calculated as the difference in dissolved organic C between fumigated and non-fumigated samples, and isotopic composition of microbial C was calculated using the equation


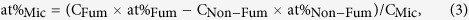


with C_Fum_ and at%_Fum_ representing content and isotopic composition of dissolved organic C in fumigated samples, and C_Non-Fum_ and at%_Non-Fum_ in non-fumigated samples. C_Mic_ and at%_Mic_ represent content and isotopic composition of microbial C. Substrate- and SOC-derived microbial C were distinguished using equations (1) and (2), replacing respired C by microbial C. The microbial efficiency to incorporate substrate-derived C was calculated by comparing substrate-derived C in the microbial biomass (MC_Substrate_) and in cumulative CO_2_ respired during the course of the incubation (RC_Substrate_) as


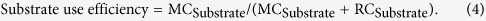


### Statistical Analyses

Statistical analyses were performed in R 2.15.0 (ref. 47), with the additional package GenABEL^48^. We applied Mann-Whitney-U tests to test for differences between horizons and Welch’s paired t-tests to test for differences between control and cellulose, control and protein, as well as cellulose and protein treatments, respectively. For paired t-tests, data were log- or rank-transformed if necessary to achieve normal distribution. We further performed Spearman’s rank sum correlations that test for a monotonous relationship between two parameters, and describe the closeness of this relationship using Spearman’s correlation coefficient rho. Differences and correlations were considered significant at p < 0.05.

Changes in SOC mineralization and microbial biomass are presented as response ratios (RR), calculated as ratios between samples amended with cellulose or protein and the respective control samples.

## Additional Information

**How to cite this article**: Wild, B. *et al.* Plant-derived compounds stimulate the decomposition of organic matter in arctic permafrost soils. *Sci. Rep.* **6**, 25607; doi: 10.1038/srep25607 (2016).

## Supplementary Material

Supplementary Information

## Figures and Tables

**Table 1:** Characterization of sampling sites.

	Coordinates	MAT ( °C)	MAP (mm)	Vegetation type	Soil type	Active layer (cm)	Dominant plant species
Cherskiy	69°26’N, 161°44’E	−12.7	160	Shrubby grass tundra	Ruptic-Histic Aquiturbel	30–70	*Betula exilis*, *Salix sphenophylla*, *Carex lugens*, *Calamagrostis holmii*, *Aulacomnium turgidum*
				Shrubby tussock tundra	Ruptic-Histic Aquiturbel	35–60	*Eriophorum vaginatum*, *Carex lugens*, *Betula exilis*, *Salix pulchra*, *Aulacomnium turgidum*
Ari-Mas	72°29’N, 101°40’E	−13.7	280	Shrubby moss tundra	Typic Aquiturbel	60–85	*Betula nana*, *Dryas punctata*, *Vaccinium uligonosum*, *Carex arctisibirica*, *Aulacomnium turgidum*
				Shrubby moss tundra	Typic Aquiturbel	65–90	*Cassiope tetragona*, *Carex arctisibirica*, *Aulacomnium turgidum*
Logata	73°26’N, 98°25’E	−13.5	270	Dryas tundra	Typic Aquiturbel	35–70	*Dryas punctata*, *Rhytidium rugosum*, *Hylocomium splendens*
				Grassy moss tundra	Typic Aquiturbel	30–65	*Betula nana*, *Carex arctisibirica*, *Hylocomium splendens*, *Tomentypnum nitens*
Tazovskiy	67°10’N, 78°55’E	−_8.2	454	Shrubby lichen tundra	Typic Aquiturbel	100–120	*Empetrum nigrum*, *Ledum palustre*, *Betula nana*, *Cladonia rangiferina*, *Cladonia stellaris*
				Forest tundra	Typic Aquiturbel	130–150	*Larix sibirica*, *Ledum palustre*, *Betula nana*, *Vaccinium uligonosum*, *Cladonia rangiferina*, *Cladonia stellaris*

Soil samples were taken from two representative vegetation types at each site. Mean annual temperature (MAT) and mean annual precipitation (MAP) were derived from the WorldClim database^51^; soil description follows the USDA Soil Taxonomy^52^. Active layer depth was determined at the time of sampling in the late growing season, the variation is due to small-scale differences in surface morphology.

**Table 2:** Characterization of the sampled soil horizons.

	Number of samples	Depth (cm)	Organic C (%)	N (%)	C/N	δ^13^C (‰)
Organic topsoils	18	10.0 ± 1.4	21.39 ± 1.46 *a*	0.87 ± 0.05 *a*	25.58 ± 1.88 *a*	−27.45 ± 0.17 *c*
Mineral topsoils	23	12.7 ± 1.5	4.17 ± 0.56 *b*	0.26 ± 0.03 *b*	15.50 ± 0.62 *b*	−27.09 ± 0.21 *c*
Mineral subsoils	29	40.6 ± 4.0	1.27 ± 0.17 *c*	0.10 ± 0.01 *c*	11.89 ± 0.49 *c*	−26.08 ± 0.27 *b*
Cryoturbated	27	46.4 ± 3.6	6.48 ± 0.92 *b*	0.37 ± 0.04 *b*	16.53 ± 0.66 *b*	−27.12 ± 0.17 *c*
Permafrost	22	89.3 ± 6.2	1.43 ± 0.44 *c*	0.09 ± 0.02 *c*	12.02 ± 1.30 *c*	−24.75 ± 0.52 *a*

Different letters indicate significant differences between horizon classes at p < 0.05. For data on individual sampling sites see Supplementary Table S1.

**Table 3:** Correlations between responses to addition of cellulose versus protein, for SOC mineralization and microbial biomass.

	RR (cellulose) vs. RR (protein): SOC mineralization	RR (cellulose) vs. RR (protein): Microbial biomass
Organic topsoils	n.s.	n.s.
Mineral topsoils	n.s.	n.s.
Mineral subsoils	0.524	0.577
Cryoturbated	n.s.	n.s.
Permafrost	0.462	0.724
All horizons	0.258	0.402

Response ratios (RR) were calculated as ratios of amended over control samples. Given values are Spearman’s rho of correlations significant at p < 0.05 (n.s., not significant).

**Table 4:** Losses of native SOC without substrate amendment, and additional losses induced by cellulose or protein input, estimated for a growing-season of four months.

	Temperature (°C)	Loss of native SOC (% of SOC)		
		No substrate amendment	Additional SOC loss induced by cellulose	Additional SOC loss induced by protein
Organic topsoils	8.0	2.23 ± 0.22	+ 0.00 ± 0.08	+ 0.89 ± 0.37
Mineral topsoils	7.5	1.65 ± 0.16	+ 0.26 ± 0.07	+ 0.55 ± 0.17
Mineral subsoils	4.5	1.33 ± 0.23	+ 0.27 ± 0.09	+ 1.13 ± 0.14
Cryoturbated	4.0	0.65 ± 0.09	+ 0.08 ± 0.04	+ 0.60 ± 0.09
Permafrost	1.0	2.67 ± 0.50	+ 0.58 ± 0.38	+ 1.09 ± 0.24

Values derived from the incubation at 15 °C were adjusted for typical growing-season soil temperatures using Q_10_ values.

## References

[b1] Bhatt U. *et al.* Circumpolar arctic tundra vegetation change is linked to sea ice decline. Earth Interact. 14, 1–20 (2010).

[b2] Xu L. *et al.* Temperature and vegetation seasonality diminishment over northern lands. Nature Clim. Change 3, 581–586 (2013).

[b3] Jones D., Nguyen C. & Finlay R. Carbon flow in the rhizosphere: carbon trading at the soil–root interface. Plant Soil 321, 5–33 (2009).

[b4] Kuzyakov Y., Friedel J. & Stahr K. Review of mechanisms and quantification of priming effects. Soil Biol. Biochem. 32, 1485–1498 (2000).

[b5] Allison S. *et al.* Low levels of nitrogen addition stimulate decomposition by boreal forest fungi. Soil Biol. Biochem. 41, 293–302 (2009).

[b6] Fontaine S. *et al.* Stability of organic carbon in deep soil layers controlled by fresh carbon supply. Nature 450, 277–280 (2007). 17994095 10.1038/nature06275

[b7] Blagodatskaya Е. & Kuzyakov Y. Mechanisms of real and apparent priming effects and their dependence on soil microbial biomass and community structure: critical review. Biol. Fert. Soils 45, 115–131 (2008).

[b8] Craine J., Morrow C. & Fierer N. Microbial nitrogen limitation increases decomposition. Ecology, 88, 2105–2113 (2007). 17824441 10.1890/06-1847.1

[b9] Dijkstra F., Carrillo Y., Pendall E. & Morgan J. Rhizosphere priming: a nutrient perspective. Front. Microbiol. 4, 216 (2013). 23908649 10.3389/fmicb.2013.00216 PMC3725428

[b10] Hartley I. *et al.* A potential loss of carbon associated with greater plant growth in the European Arctic. Nature Clim. Change, 2, 875–879 (2012).

[b11] Sistla S. *et al.* Long-term warming restructures Arctic tundra without changing net soil carbon storage. Nature 497, 615–618 (2013). 23676669 10.1038/nature12129

[b12] Lavoie M., Mack M. & Schuur E. Effects of elevated nitrogen and temperature on carbon and nitrogen dynamics in Alaskan arctic and boreal soils. J. Geophys. Res. 116, G03013 (2011).

[b13] Sistla S., Asao S. & Schimel J. Detecting microbial N-limitation in tussock tundra soil: Implications for Arctic soil organic carbon cycling. Soil Biol. Biochem. 55, 78–84 (2012).

[b14] Hartley I., Hopkins D., Sommerkorn M. & Wookey P. The response of organic matter mineralisation to nutrient and substrate additions in sub-arctic soils. Soil Biol. Biochem. 42, 92–100 (2010).

[b15] Wild B. *et al.* Input of easily available organic C and N stimulates microbial decomposition of soil organic matter in arctic permafrost soil. Soil Biol. Biochem. 75, 143–151 (2014). 25089062 10.1016/j.soilbio.2014.04.014 PMC4064687

[b16] Ping C. L., Jastrow J. D., Jorgenson M. T., Michaelson G. J. & Shur Y. L. Permafrost soils and carbon cycling. SOIL 1, 147–171 (2015).

[b17] Kaiser C. *et al.* Conservation of soil organic matter through cryoturbation in arctic soils in Siberia. J. Geophys. Res. 112, G02017 (2007).

[b18] Hugelius G. *et al.* Estimated stocks of circumpolar permafrost carbon with quantified uncertainty ranges and identified data gaps. Biogeosciences 11, 6573–6593 (2014).

[b19] Lawrence D. M., Slater A. G. & Swenson S. C. Simulation of present-day and future permafrost and seasonally frozen ground conditions in CCSM4. J. Climate 25, 2207–2225 (2012).

[b20] Ernakovich J. G. *et al.* Predicted responses of arctic and alpine ecosystems to altered seasonality under climate change. Glob. Change Biol. 20, 3256–3269 (2014). 10.1111/gcb.12568 24599697

[b21] Schuur E. A. G. *et al.* Climate change and the permafrost carbon feedback. Nature 520, 171–179 (2015). 25855454 10.1038/nature14338

[b22] Wild B. *et al.* Nitrogen dynamics in Turbic Cryosols from Siberia and Greenland. Soil Biol. Biochem. 67, 85–93 (2013). 24302785 10.1016/j.soilbio.2013.08.004 PMC3819518

[b23] Mooshammer M. *et al.* Adjustment of microbial nitrogen use efficiency to carbon:nitrogen imbalances regulates soil nitrogen cycling. Nat. Commun. 5, 3694 (2014). 24739236 10.1038/ncomms4694 PMC3997803

[b24] Koyama A., Wallenstein M., Simpson R. & Moore J. Carbon-degrading enzyme activities stimulated by increased nutrient availability in arctic tundra soils. PLos ONE 8, e77212 (2013). 24204773 10.1371/journal.pone.0077212 PMC3817314

[b25] Weintraub M. & Schimel J. Seasonal protein dynamics in Alaskan arctic tundra soils. Soil Biol. Biochem. 37, 1469–1475 (2005).

[b26] Karhu K. *et al.* Temperature sensitivity of soil respiration rates enhanced by microbial community response. Nature 513, 81–84 (2014). 25186902 10.1038/nature13604

[b27] German D., Marcelo K., Stone M. & Allison S. The Michaelis-Menten kinetics of soil extracellular enzymes in response to temperature: a cross-latitudinal study. Glob. Change Biol. 18, 1468–1479 (2012).

[b28] Schimel J., Bilbrough C. & Welker J. Increased snow depth affects microbial activity and nitrogen mineralization in two Arctic tundra communities. Soil Biol. Biochem. 36, 217–227 (2004).

[b29] Schaeffer S., Sharp E., Schimel J. & Welker J. Soil-plant N processes in a High Arctic ecosystem, NW Greenland are altered by long-term experimental warming and higher rainfall. Glob. Change Biol. 19, 3529–3539 (2013). 10.1111/gcb.12318 23843128

[b30] Gentsch N. *et al.* Storage and transformation of organic matter fractions in cryoturbated permafrost soils across the Siberian Arctic. Biogeosciences 12, 4525–4542 (2015).

[b31] Sullivan P. & Welker J. Warming chambers stimulate early season growth of an arctic sedge: results of a minirhizotron field study. Oecologia 142, 616/626 (2005). 15688218 10.1007/s00442-004-1764-3

[b32] Deslippe J. & Simard S. Below-ground carbon transfer among *Betula nana* may increase with warming in Arctic tundra. New Phytol. 192, 689–698 (2011). 21797881 10.1111/j.1469-8137.2011.03835.x

[b33] MacDougall A., Avis C. & Weaver A. Significant contribution to climate warming from the permafrost carbon feedback. Nature Geosci. 5, 719–721 (2012).

[b34] Harden J. *et al.* Field information links permafrost carbon to physical vulnerabilities of thawing. Geophys. Res. Lett. 39, L15704 (2012).

[b35] Ciais P. *et al.* Climate Change 2013: The Physical Science Basis. Contribution of Working Group I to the Fifth Assessment Report of the Intergovernmental Panel on Climate Change (eds Stocker T. F. *et al.*) Ch. 6, 456–570 (Cambridge University Press, 2013).

[b36] Jackson R. B. *et al.* A global analysis of root distributions for terrestrial biomes. Oecologia 108, 389–411 (1996). 10.1007/BF00333714 28307854

[b37] Biasi C. *et al.* Initial effects of experimental warming on carbon exchange rates, plant growth and microbial dynamics of a lichen-rich dwarf shrub tundra in Siberia. Plant Soil 307, 191–205 (2008).

[b38] Natali S. M., Schuur E. A. G., Webb E. E., Hicks Pries C. E. & Crummer K. G. Permafrost degradation stimulates carbon loss from experimentally warmed tundra. Ecology 95, 602–608 (2014). 24804439 10.1890/13-0602.1

[b39] Parker T. C., Subke J.-A. & Wookey P. A. Rapid carbon turnover beneath shrub and tree vegetation is associated with low soil carbon stocks at a subarctic treeline. Glob. Change Biol. 21, 2070–2081 (2015). 10.1111/gcb.12793 PMC4657486 25367088

[b40] Degens B. P. Decreases in microbial functional diversity do not result in corresponding changes in decomposition under different moisture conditions. Soil Biol. Biochem. 30, 1989–2000 (1998).

[b41] Degens B. P., Schipper L. A., Sparling G. P. & Duncan L. C. Is the microbial community in a soil with reduced catabolic diversity less resistant to stress or disturbance? Soil Biol. Biochem. 33, 1143–1153 (2001).

[b42] Fierer N. & Schimel J. P. Effects of drying-rewetting frequency on soil carbon and nitrogen transformations. Soil Biol.Biochem. 34, 777–787 (2002).

[b43] Fierer N., Schimel J. P. & Holden P. A. Influence of drying-rewetting frequency on soil bacterial community structure. Microbial Ecol. 45, 63–71 (2003). 10.1007/s00248-002-1007-2 12469245

[b44] Sparling G. & West A. A comparison of gas-chromatography and differential respirometer methods to measure soil respiration and to estimate the soil microbial biomass. Pedobiologia 34, 103–112 (1990).

[b45] Brookes P. C., Landman A., Pruden G. & Jenkinson D. S. Chloroform fumigation and the release of soil nitrogen: A rapid direct extraction method to measure microbial biomass nitrogen in soil. Soil Biol. Biochem. 17, 837–842 (1985).

[b46] Wild B., Wanek W., Postl W. & Richter A. Contribution of carbon fixed by Rubisco and PEPC to phloem export in the Crassulacean acid metabolism plant *Kalanchoe daigremontiana*. J. Exp. Bot. 61, 1375–1383 (2010). 20159885 10.1093/jxb/erq006 PMC2837257

[b47] Core Team R. R: A language and environment for statistical computing. R Foundation for Statistical Computing, Vienna, Austria. URL http://www.R-project.org/ (2012).

[b48] Aulchenko Y., Ripke S., Isaacs A. & Duijn C. GenABEL: an R library for genome-wide association analysis. Bioinformatics 23, 1294–1296 (2007). 17384015 10.1093/bioinformatics/btm108

[b49] Pebesma E. J. & Bivand R. S. Classes and methods for spatial data in R. R News 5, 9–13 (2005).

[b50] South A. rworldmap: a new R package for mapping global data. The R Journal 3, 35–43 (2011).

[b51] Hijmans R., Cameron S., Parra J., Jones P. & Jarvis A. Very high resolution interpolated climate surfaces for global land areas. Int. J. Climatol. 25, 1965–1978 (2005).

[b52] Soil Survey Staff. Keys to soil taxonomy, 12^th^ ed. (USDA-Natural Resources Conservation Service, 2014).

