# Supplementary material for: Plant-derived compounds stimulate the decomposition of organic matter in arctic permafrost soils
Source: Sci Rep. 2016 May 9;6:25607. doi: 10.1038/srep25607 (PMC4860603; doi:10.1038/srep25607)
Supplement: Supplementary Information [file srep25607-s1.pdf]

Plant-derived compounds stimulate the decomposition of organic matter in arctic permafrost soils

Birgit Wild[1,2,3]*, Norman Gentsch[4], Petr Čapek[5], Kateřina Diáková[5], Ricardo J. Eloy Alves[2,6], Jiři Bárta[5], Antje Gittel[7,8], Gustaf Hugelius[9], Anna Knoltsch[1,2], Peter Kuhry[9], Nikolay Lashchinskiy[10], Robert Mikutta[4,11], Juri Palmtag[9], Christa Schleper[2,6], Jörg Schnecker[1,2,12], Olga Shibistova[4,13], Mounir Takriti[1,2,14], Vigdis L. Torsvik[7], Tim Urich[2,6,15], Margarete Watzka[1], Hana Šantrůčková[5], Georg Guggenberger[4,13], Andreas Richter[1,2]*

[1] Department of Microbiology and Ecosystem Science, University of Vienna, Vienna, Austria

[2] Austrian Polar Research Institute, Vienna, Austria

[3] Department of Earth Sciences, University of Gothenburg, Gothenburg, Sweden

[4] Institute of Soil Science, Leibniz Universität Hannover, Hannover, Germany

[5] Department of Ecosystem Biology, University of South Bohemia, České Budějovice, Czech Republic

[6] Department of Ecogenomics and Systems Biology, University of Vienna, Vienna, Austria

[7] Department of Biology, Centre for Geobiology, University of Bergen, Bergen, Norway

[8] Department of Bioscience, Center for Geomicrobiology, Aarhus, Denmark

[9] Department of Physical Geography, Stockholm University, Stockholm, Sweden

[10] Central Siberian Botanical Garden, Siberian Branch of Russian Academy of Sciences, Novosibirsk, Russia

[11] Soil Science and Soil Protection, Martin-Luther-University Halle-Wittenberg, Halle (Saale), Germany

[12] Department of Natural Resources and the Environment, University of New Hampshire, Durham, NH, USA

[13] VN Sukachev Institute of Forest, Siberian Branch of Russian Academy of Sciences, Krasnoyarsk, Russia

[14] Lancaster Environment Centre, Lancaster University, Lancaster, UK

[15] Institute of Microbiology, Ernst-Moritz-Arndt University, Greifswald, Germany

* Corresponding authors:

Birgit Wild, +46 31 786 2828, birgit.wild@gu.se

Andreas Richter, +43 1 4277 76660, andreas.richter@univie.ac.at

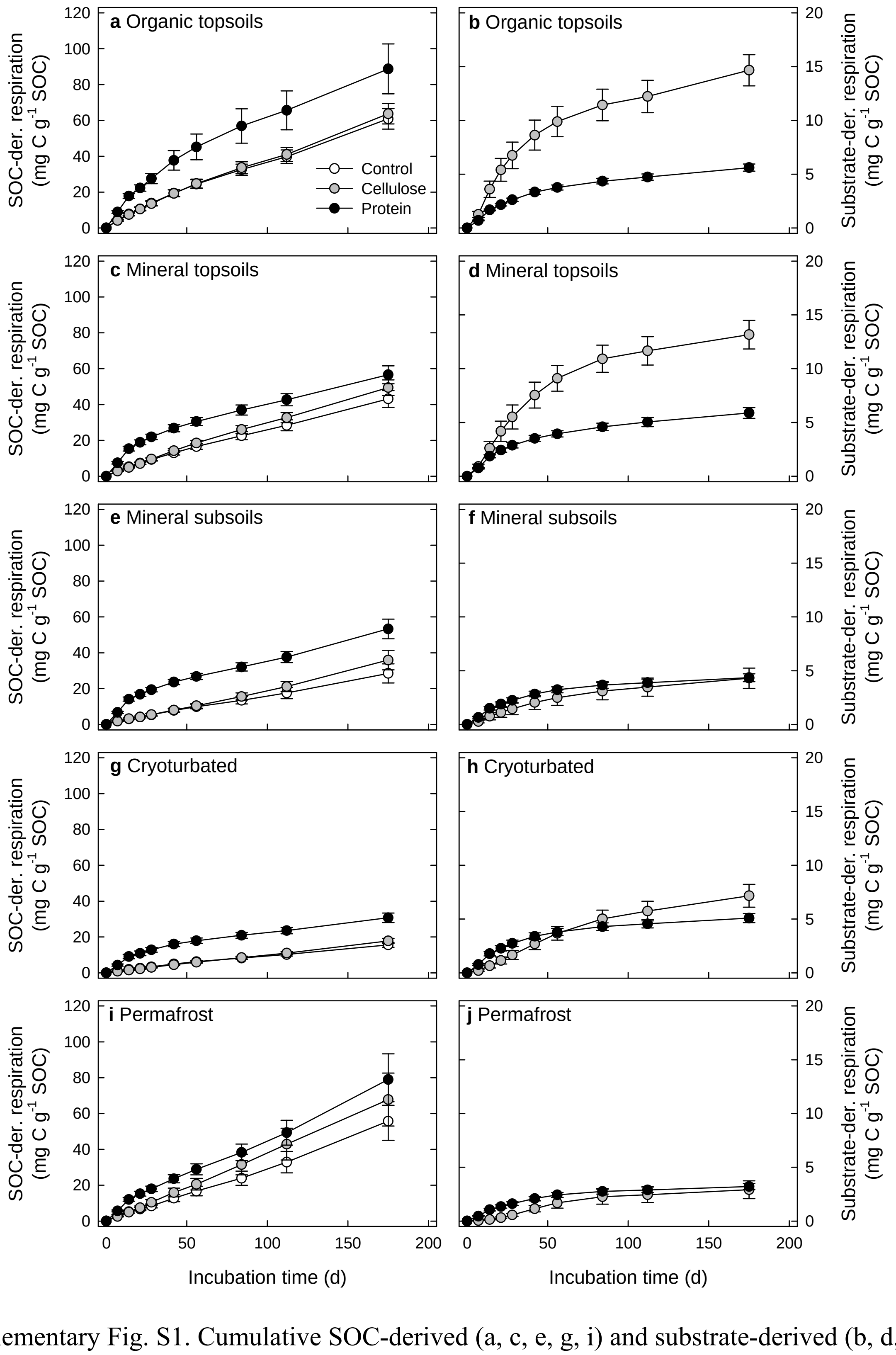

Supplementary Fig. S1. Cumulative SOC-derived (a, c, e, g, i) and substrate-derived (b, d, f, h, j) respiration over 25 weeks of incubation in different horizons of arctic permafrost soils after addition of cellulose or protein, and in unamended control samples. Values represent means with standard errors. Note the differences in scaling.

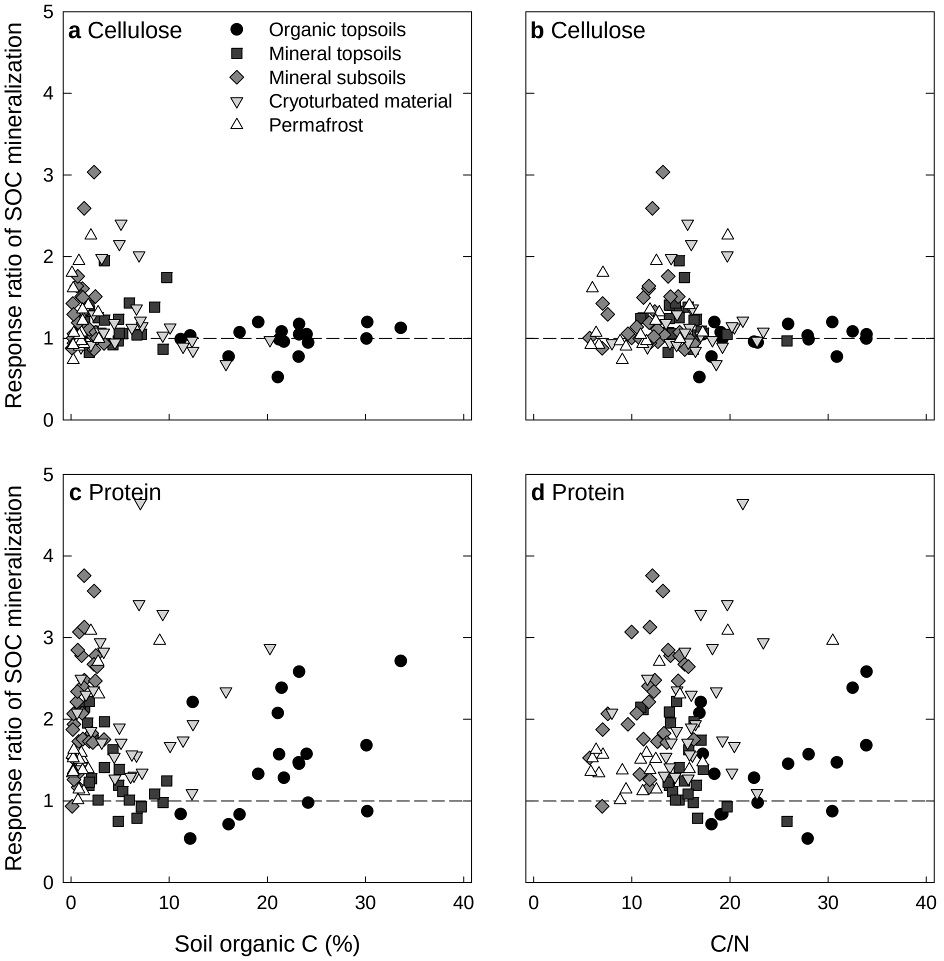

Supplementary Fig. S2. Responses of cumulative SOC mineralization to addition of cellulose or protein, plotted against SOC content and C/N ratio. Response ratios were calculated as ratios of samples amended with cellulose or protein over control samples. Note the threshold of circa 10% SOC and a C/N ratio of 20, above which positive responses to cellulose are negligible.

Supplementary Table S1. Characterization of the sampled soil horizons, individually for the four sites across the Siberian Arctic.

	Number of samples	Depth (cm)	Organic C (%)	N (%)	C/N	$\delta^{13}C$ (‰)
Organic topsoils	18	10.0 ± 1.4	21.39 ± 1.46	0.87 ± 0.05	25.58 ± 1.88	-27.45 ± 0.17
Cherskiy	7	11.8 ± 2.8	20.80 ± 1.56	1.05 ± 0.08	20.11 ± 1.36	-27.98 ± 0.10
Ari-Mas	3	12.5 ± 5.0	17.83 ± 6.19	0.67 ± 0.17	25.86 ± 3.39	-27.89 ± 0.12
Logata	2	7.5 ± 0.0	16.61 ± 0.56	0.89 ± 0.01	18.58 ± 0.47	-27.33 ± 0.50
Tazovskiy	6	7.5 ± 0.0	25.46 ± 2.10	0.75 ± 0.03	34.50 ± 2.50	-26.65 ± 0.21
Mineral topsoils	23	12.7 ± 1.5	4.17 ± 0.56	0.26 ± 0.03	15.50 ± 0.62	-27.09 ± 0.21
Cherskiy	4	15.0 ± 4.3	1.47 ± 0.19	0.12 ± 0.01	12.64 ± 0.94	-26.67 ± 0.25
Ari-Mas	7	18.2 ± 2.8	3.84 ± 0.71	0.22 ± 0.03	17.04 ± 1.66	-27.57 ± 0.22
Logata	7	9.6 ± 2.1	7.12 ± 0.80	0.49 ± 0.05	15.49 ± 0.35	-27.82 ± 0.25
Tazovskiy	5	7.5 ± 0.0	2.67 ± 0.68	0.17 ± 0.04	15.64 ± 0.78	-25.70 ± 0.09
Mineral subsoils	29	40.6 ± 4.0	1.27 ± 0.17	0.10 ± 0.01	11.89 ± 0.49	-26.08 ± 0.27
Cherskiy	8	41.6 ± 4.7	1.50 ± 0.21	0.12 ± 0.01	12.04 ± 0.52	-27.04 ± 0.28
Ari-Mas	6	45.0 ± 6.4	0.63 ± 0.12	0.05 ± 0.01	11.77 ± 0.61	-25.88 ± 0.36
Logata	8	24.4 ± 6.0	2.29 ± 0.22	0.16 ± 0.01	14.12 ± 0.62	-26.91 ± 0.29
Tazovskiy	7	54.6 ± 11.1	0.39 ± 0.09	0.04 ± 0.01	9.28 ± 1.20	-24.10 ± 0.37
Cryoturbated	27	46.4 ± 3.6	6.48 ± 0.92	0.37 ± 0.04	16.53 ± 0.66	-27.12 ± 0.17
Cherskiy	4	48.8 ± 3.8	14.45 ± 2.34	0.82 ± 0.12	17.58 ± 0.50	-27.49 ± 0.31
Ari-Mas	9	60.8 ± 7.5	3.55 ± 0.62	0.22 ± 0.04	16.18 ± 0.95	-27.50 ± 0.16
Logata	8	39.4 ± 3.4	8.02 ± 1.02	0.45 ± 0.03	17.47 ± 1.29	-27.46 ± 0.10
Tazovskiy	6	32.5 ± 5.0	3.50 ± 1.17	0.20 ± 0.05	15.09 ± 2.03	-25.92 ± 0.32
Permafrost	22	89.3 ± 6.2	1.43 ± 0.44	0.09 ± 0.02	12.02 ± 1.30	-24.75 ± 0.52
Cherskiy	6	67.5 ± 3.9	2.91 ± 1.28	0.16 ± 0.03	14.58 ± 3.31	-26.88 ± 0.48
Ari-Mas	5	82.5 ± 4.7	1.42 ± 0.29	0.09 ± 0.01	16.34 ± 1.52	-23.63 ± 2.04
Logata	5	70.5 ± 5.6	1.06 ± 0.08	0.09 ± 0.00	11.67 ± 0.32	-24.44 ± 0.29
Tazovskiy	6	132.5 ± 3.2	0.20 ± 0.03	0.03 ± 0.00	6.81 ± 0.48	-23.58 ± 0.33

Supplementary Table S2. Percentage of samples showing response ratios of SOC mineralization higher than 1.2 or lower than 0.8 after addition of cellulose or protein. Response ratios were calculated as ratios of amended over control samples.

	Response ratio > 1.2		Response ratio < 0.8	
	Cellulose	Protein	Cellulose	Protein
Organic topsoils	0.0%	66.7%	17.6%	11.1%
Mineral topsoils	56.5%	56.5%	0.0%	8.7%
Mineral subsoils	40.7%	93.1%	0.0%	0.0%
Cryoturbated	32.0%	96.3%	4.0%	0.0%
Permafrost	42.9%	81.8%	4.8%	0.0%
All horizons	36.3%	80.7%	4.4%	3.4%

Supplementary Table S3. Microbial biomass C after 25 weeks of incubation, per dry soil and per soil organic C. Different letters indicate significant differences between horizon classes at $p < 0.05$.

	Microbial biomass C per dry soil ($\mu g\ C\ g^{-1}$ dry soil)	Microbial biomass C per soil organic C ($mg\ C\ g^{-1}$ SOC)
Organic topsoils	849.60 ± 106.27 *a*	3.95 ± 0.39 *a*
Mineral topsoils	174.74 ± 41.50 *b*	3.39 ± 0.50 *a*
Mineral subsoils	48.98 ± 5.76 *c*	4.99 ± 0.84 *a*
Cryoturbated	126.60 ± 18.93 *b*	2.11 ± 0.21 *b*
Permafrost	50.00 ± 7.74 *c*	6.66 ± 1.27 *a*

Supplementary Table S4. Spearman's rho of correlations between SOM C/N ratios, microbial substrate use efficiencies (SUE) of cellulose- or protein-derived C, and the response ratios of SOC mineralization after cellulose or protein input (RR; calculated as ratios of amended over control samples). n.s., no significant correlation at $p < 0.05$.

	Cellulose			Protein		
	C/N vs. SUE	C/N vs. RR	SUE vs. RR	C/N vs. SUE	C/N vs. RR	SUE vs. RR
Organic topsoils	n.s.	n.s.	n.s.	-0.554	n.s.	n.s.
Mineral topsoils	n.s.	n.s.	n.s.	n.s.	-0.472	n.s.
Mineral subsoils	n.s.	n.s.	n.s.	n.s.	n.s.	-0.455
Cryoturbated	-0.468	n.s.	n.s.	n.s.	n.s.	n.s.
Permafrost	n.s.	n.s.	n.s.	n.s.	n.s.	n.s.
All horizons	-0.481	n.s.	n.s.	-0.343	n.s.	n.s.

Supplementary Table S5. Responses of native SOC mineralization and microbial biomass in different horizons of arctic permafrost soils to the addition of cellulose or protein. Response ratios were calculated as ratios of amended over control samples. Values represent means ± standard errors, bold values indicate significant differences in SOC mineralization between amended and control samples at $p < 0.05$ (Welch's paired t-test).

	Response ratio of SOC mineralization		Response ratio of the microbial biomass	
	Cellulose	Protein	Cellulose	Protein
Organic topsoils	1.00 ± 0.04	**1.51 ± 0.16**	1.07 ± 0.10	1.20 ± 0.13
Cherskiy	0.97 ± 0.10	**1.56 ± 0.17**	0.99 ± 0.18	1.34 ± 0.19
Ari-Mas	1.07 ± 0.06	**0.75 ± 0.11**	1.29 ± 0.26	0.89 ± 0.26
Logata	0.92 ± 0.15	0.77 ± 0.06	0.97 ± 0.54	1.38 ± 0.21
Tazovskiy	1.00 ± 0.05	**2.07 ± 0.23**	1.09 ± 0.07	1.11 ± 0.32
Mineral topsoils	**1.22 ± 0.06**	**1.41 ± 0.10**	**1.64 ± 0.20**	**1.57 ± 0.30**
Cherskiy	1.31 ± 0.04	**2.11 ± 0.06**	0.94 ± 0.24	2.88 ± 1.42
Ari-Mas	1.27 ± 0.13	1.11 ± 0.08	2.04 ± 0.36	1.27 ± 0.28
Logata	1.21 ± 0.12	1.12 ± 0.10	1.36 ± 0.18	1.14 ± 0.06
Tazovskiy	1.09 ± 0.07	**1.68 ± 0.17**	2.06 ± 1.08	1.52 ± 0.59
Mineral subsoils	**1.31 ± 0.10**	**2.20 ± 0.13**	1.56 ± 0.28	1.53 ± 0.30
Cherskiy	**1.75 ± 0.29**	**2.87 ± 0.21**	1.39 ± 0.85	1.69 ± 0.92
Ari-Mas	1.11 ± 0.09	**1.70 ± 0.25**	2.17 ± 0.79	2.10 ± 0.47
Logata	1.08 ± 0.08	**2.11 ± 0.17**	1.63 ± 0.25	1.10 ± 0.33
Tazovskiy	1.29 ± 0.13	**1.97 ± 0.23**	0.69 ± 0.21	1.04 ± 0.68
Cryoturbated	**1.22 ± 0.09**	**2.09 ± 0.16**	1.34 ± 0.14	**1.48 ± 0.16**
Cherskiy	0.89 ± 0.08	**2.61 ± 0.30**	0.65 ± 0.24	1.30 ± 0.16
Ari-Mas	**1.50 ± 0.18**	**1.91 ± 0.18**	1.49 ± 0.26	1.37 ± 0.16
Logata	1.08 ± 0.05	**1.45 ± 0.08**	**1.71 ± 0.14**	1.16 ± 0.16
Tazovskiy	1.17 ± 0.18	**2.89 ± 0.42**	0.94 ± 0.32	2.29 ± 0.55
Permafrost	1.23 ± 0.09	**1.63 ± 0.12**	1.14 ± 0.20	1.28 ± 0.22
Cherskiy	1.02 ± 0.08	1.87 ± 0.36	0.93 ± 0.27	1.15 ± 0.43
Ari-Mas	**1.63 ± 0.21**	**1.71 ± 0.35**	1.05 ± 0.25	1.16 ± 0.13
Logata	1.10 ± 0.08	1.48 ± 0.04	1.12 ± 0.39	1.47 ± 0.63
Tazovskiy	1.17 ± 0.17	**1.46 ± 0.05**	1.64 ± 0.87	1.33 ± 0.44

Supplementary Table S6. Microbial substrate use efficiency of cellulose- or protein-derived C in different horizons of arctic permafrost soils. Substrate use efficiency was calculated as the ratio of substrate-derived C in microbial biomass over substrate-derived C in biomass plus cumulative respiration after 25 weeks of incubation. Values represent means ± standard errors.

	Substrate use efficiency	
	Cellulose	Protein
Organic topsoils	0.048 ± 0.006	0.036 ± 0.004
Cherskiy	0.041 ± 0.011	0.036 ± 0.004
Ari-Mas	0.045 ± 0.017	0.030 ± 0.006
Logata	0.054 ± 0.011	0.059 ± 0.010
Tazovskiy	0.056 ± 0.007	0.031 ± 0.009
Mineral topsoils	0.064 ± 0.006	0.041 ± 0.004
Cherskiy	0.077 ± 0.012	0.045 ± 0.011
Ari-Mas	0.069 ± 0.012	0.045 ± 0.006
Logata	0.055 ± 0.010	0.050 ± 0.006
Tazovskiy	0.055 ± 0.012	0.021 ± 0.003
Mineral subsoils	0.132 ± 0.019	0.058 ± 0.006
Cherskiy	0.083 ± 0.019	0.067 ± 0.012
Ari-Mas	0.152 ± 0.028	0.078 ± 0.008
Logata	0.108 ± 0.014	0.044 ± 0.004
Tazovskiy	0.208 ± 0.075	0.028 ± 0.020
Cryoturbated	0.080 ± 0.010	0.046 ± 0.005
Cherskiy	0.048 ± 0.006	0.055 ± 0.022
Ari-Mas	0.091 ± 0.018	0.053 ± 0.009
Logata	0.098 ± 0.017	0.043 ± 0.004
Tazovskiy	0.052 ± 0.012	0.029 ± 0.007
Permafrost	0.199 ± 0.040	0.121 ± 0.018
Cherskiy	0.235 ± 0.096	0.100 ± 0.034
Ari-Mas	0.112 ± 0.051	0.077 ± 0.025
Logata	0.233 ± 0.060	0.210 ± 0.017
Tazovskiy	0.242 ± 0.183	0.082 ± 0.019
